# Tuberculous Abscess of the Lungs Opening Externally

**Published:** 1846-11

**Authors:** 


*Tuberculous Abscess of the Lungs opening externally.*—M. Forget, of Strasburg, read at the Academy of Medicine in Paris, the details of a patient affected with pulmonary phthisis, in whom there was developed on one side of the chest a fluctuating tumor, with emphysematous crepitation. This tumor opened spontaneously, and gave exit to both pus and air; a fistula, which closed and again opened at different times, persisted to the death of the patient, which was accelerated by the appearance of a plueritic effusion occupying the entire thoracic cavity. At the autopsy, a tuberculous caries of the seventh and eighth ribs, corresponding to the abscess, and a fistulous opening establishing a communication between the exterior of the chest, and the inte ior of the lung appeared. Tubercles existed in the lungs, and liquid and false membranes in the pleura.

M. Velpeau observed to M. Forget, who believes this case unique, that he possessed himself six analogous to it, two of which were even still more curious, for the fistula opened on the lateral parities of the neck, below the ear.

M. Blandin related likewise three facts similar to that of M. Forget. He spoke of tuberculous abscesses communicating with the lung, which he had opened in the practice of Messrs. L'Herminier, Honoré, Rostan.—*Gaz. Med. Chir.—Med. News.*

